# Natural Food Resource Valorization by Microwave Technology: Purslane Stabilization by Dielectric Heating

**DOI:** 10.3390/foods12234247

**Published:** 2023-11-24

**Authors:** Marco Apicella, Giuseppe Amato, Pietro de Bartolomeis, Anna Angela Barba, Vincenzo De Feo

**Affiliations:** 1 Department of Pharmacy, University of Salerno, Via Giovanni Paolo II 132, 84084 Fisciano, Italy; mapicella@unisa.it (M.A.); gamato@unisa.it (G.A.); defeo@unisa.it (V.D.F.); 2 Caselle Società Agricola Srl, Via Mare Mediterraneo 18, 84098 Pontecagnano, Italy; info@elody.it; 3 EST Srl, University Spin-Off, Via Circumvallazione n.39, 83100 Avellino, Italy; 4 Eng4Life Srl, Via Circumvallazione n.39, 83100 Avellino, Italy

**Keywords:** *Portulaca oleracea* L., fatty acids, phytosterols, dielectric heating, microwave drying technology, food recovery, valorization, future food

## Abstract

The application of microwave-assisted drying is a promising technique due to the features of process sustainability that are usable for responsible productions. It is largely applied for the stabilization of food products, especially in the agro-food sector. In this study, the weed *Portulaca oleracea* L. (purslane), with its richness in antioxidant components in addition to its recognized pharmacological properties, has been considered due to its potential to be a natural, well-accepted future food. Attention was focused on the role of the heat and mass transfer rates involved in the drying processes on the nutritional profile of the dried products. For this purpose, different drying protocols (convective, microwave irradiation, microwave-vacuum irradiation) were applied to different parts of purslane herb (apical, twigs, entire structures) and chemical characterizations were performed by a GC/MS analysis of the extracts of the dried products. The results show that microwave treatments can assure a better preservation of fatty acids such as SFAs, MUFAs, and PUFAs (which constitute over 90% of the total components in the apical part, 65% in twigs, and 85% in microwave-vacuum-dried entire purslane samples) and phytosterols (their highest preservation was found in microwave-dried twigs) compared with convective treatments. The chemical composition variability as well as treatment times depend on the drying rates (in microwave treatments, the times are on a minute scale and the rates are up to three orders of magnitude greater than convective ones), which in turn depend on the heating transport phenomena. This variability can lead towards products that are diversified by properties that transform a weed into a valorized food source.

## 1. Introduction

Valorization of natural products for food purposes is one of the main challenges for ensuring sustainable consumption and production patterns, and it is essential for supporting the livelihoods of current and future generations [1,2]. Sustainable consumption and production models are strategies that are currently being promoted to reduce poverty and hunger and prevent climate change, biodiversity loss, and pollution. The vegetable kingdom offers the possibility of using innumerable crops, both wild and cultivated, to promote policies of sustainability by exploiting renewable sources for the global food supply.

Among the natural assets, purslane *Portulaca oleracea* L., a wild edible plant (WEP), has received great attention in recent years for several reasons [3]. It is a widespread vegetal species that is wholly edible and can be eaten raw without toxicity effects or can be cooked or pickled [4]. Purslane has been defined as a “power food” thanks to its high nutritional and antioxidant properties, and it is listed by the World Health Organization as one of the most useful medicinal plants, giving it the title of “global panacea” [5]. It is able to grow in natural conditions without human intervention and is traditionally used as a food source or as a complementary ingredient in local recipes, or even as “famine food” [3]. In particular, purslane is a ubiquitous weed worldwide; it is commonly known as “porcelain” and is the eighth most common plant in the world [6]. Purslane belongs to the Portulacaceae family, many species of which are cultivated as herbs or as ornamental bedding plants. This species is annual and grows during the warmest months of the year due to its ability to stock water, thus also surviving in arid conditions. The exact origin of this weed is unknown; it is thought to have originated in Iran [7], but it has also been considered to be native to the Himalayan Mountains, South Russia, and India [8]. Its presence has been proven to be in America since pre-Columbian times and in Egypt and Italy during the Roman Age, thanks to the discovery of two sub-species during archaeological excavations [9]. Its exact diffusion routes were not elucidated. Purslane is generally considered as an infestant due its growth during vegetable cultivations; it also largely presents itself as a spontaneous species in lawns and landscapes.

**Figure 1:**
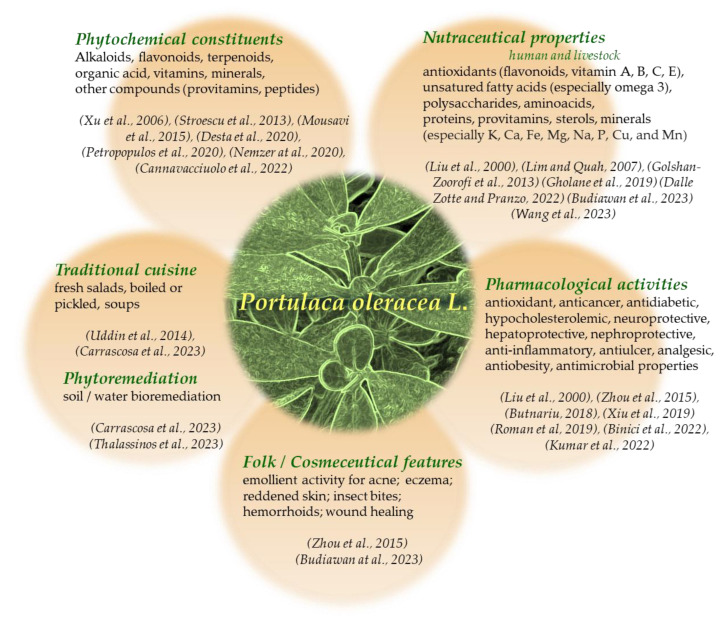
*Portulaca oleracea* L. phytochemical constituents, uses, and benefits [3,4,5,7,8,10,11,12,13,14,15,16,17,18,20,21,22,23,24,25,27].

Purslane has been extensively studied under a phytochemical point of view with the aim of providing scientific value to its folk (and ancient) medicinal uses and understanding the bases of nutritive role factors and pharmacological properties [5,10,11,12,13]. The presence of polysaccharides, flavonoids, alkaloids, fatty acids, terpenoids, sterols, proteins, vitamins, and minerals has been detected in this plant [7,11,14,15,16,17,18]. These constituents give purslane a high nutritional value, directing it towards the production of food supplements on an industrial scale. Many research studies are also underway on purslane as feed for farm animals for positive actions on reproductive factors [19,20] and the quality of farm products [21]. Furthermore, the plant possesses a wide spectrum of pharmacological properties, such as neuroprotective, antioxidant, anticancer, antidiabetic, hypocholesterolemic, hepatoprotective, nephroprotective, anti-inflammatory, antiulcer, analgesic, antiobesity, antimicrobial, and wound-healing activities [8,10,11,22,23,24,25,26]. Efficacious use of purslane in phytoremediation applications is also proven [3,27]. Figure 1 summarizes the phytochemical components, uses, and nutritional and pharmacological activities of purslane along with recent literature references.

In this study, spontaneous *P. oleracea* was used as an exploitable natural food resource, and different drying methods were applied as stabilizing treatments. In particular, for its above-mentioned richness in antioxidant components, purslane has been considered due to its potential to be cultivated (or harvested from wild lands) as a food ingredient for nutraceutical applications. Drying is the most common preservation method used in the agri-food sector [28]; the quality of dried products is strongly dependent on the technology and process variables used. The main goal is the reduction of water activity by moisture removal; this action is also accomplished with a significant reduction of volume and weight, minimizing packaging, transportation, and storage costs. Drying also alters other physical (hardness, aroma, palatability), biological (microbial spoilage), and chemical (nutritive components) properties of foods. Thus, suitable technology has to be involved [28]. Drying involves the simultaneous transfer of heat, sssmass, and momentum wherein heat penetrates into the product and moisture is moved into an unsaturated gas phase. Owing to the complexity of the process, it is not trivial to explain the mechanisms of internal moisture movement that is recognized as the major rate-limiting step in the drying process [29,30].

On an industrial scale, convective hot-air drying is one of the most effective ways for food dehydration. A major disadvantage associated with this treatment is that it takes a long time, even at high temperature, which may cause serious damage to the flavor, color, and nutrients in dried products [31]. Sun drying is limited to particular folk practices and non-industrialized applications; its disadvantages include the inability to control the process (including low-energy efficiency), handle large quantities, or achieve products with a standard final quality [32].

Microwave technology (MT) has been widely experienced as an alternative, reliable approach for power application uses. In the agricultural sector (foods and herbs), many research studies have been conducted [30,33,34]. MT is based on a unique heating mode facilitated by electromagnetic radiation at 915 MHz or 2.45 GHz. Microwave energy is directly delivered to materials through “loss mechanisms” that allow for the conversion of electromagnetic energy into thermal energy. This action represents the turning point of the technology because it makes it possible to improve the energy efficiency. Moreover, benefits for process equipment (reduced mechanical stress, compact treatment camera) and materials (selective and volumetric heating, secondary effect on tissue structure/metabolites, microbial spoilage) are also achievable [33,34]. The recent increase in interest is occurring hand-in-hand with the advances in mechanized industry and electronic communication, which are base elements of the actual industrial revolution well known as Industry 4.0. In the food processing industry, it has become significant to improve the energy efficiency and replace the existing energy-intensive unit operations such as drying with new energy-efficient processes [35,36].

In this study, the effectiveness of microwave technology for purslane stabilization purposes was investigated. Due to the plant’s extremely heterogeneous vegetal structures and high moisture content, purslane drying treatments are not trivial. Different thermal behaviors of tiny and coarse parts impact process effectiveness and the final quality of the dried structures. For these reasons, purslane weed underwent different drying protocols to analyze the effects of different rates of involved transport phenomena and their role on the better preservation of the nutritional qualities of dried products.

## 2. Materials and Methods

### 2.1. Purslane Supply

**Figure 2:**
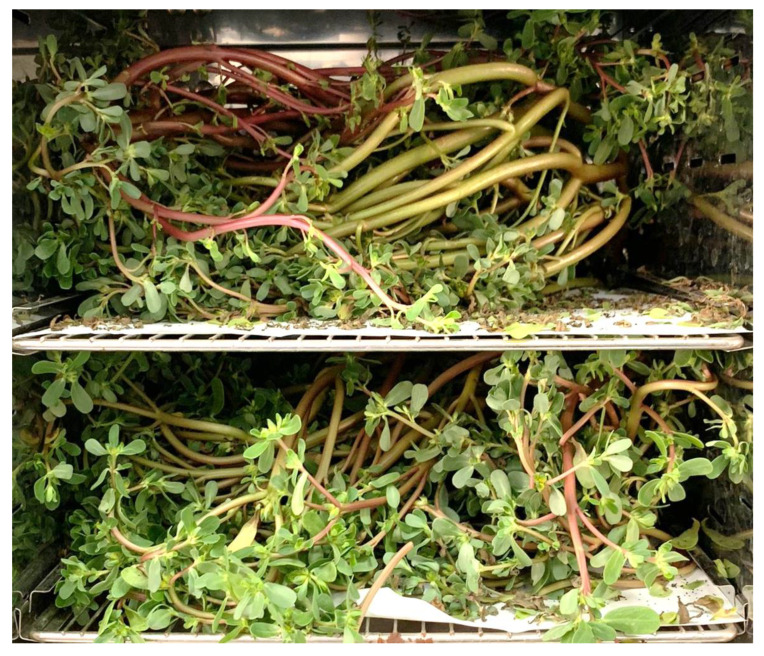
*Portulaca oleracea* fresh specimens.

Purslane (*Portulaca oleracea* L.) was collected at the farm of the agricultural company Caselle Società Agricola (site in Pontecagnano, Salerno, Italy) in July 2021 (June–July 2021; average temperatures and humidity: 26.6–28.8 °C; 53 RU%, respectively) from a natural spontaneous population (GPS: 40°63′77.2” N, 14°86′59” E). The plant was identified by Prof. Vincenzo De Feo. A voucher specimen, labelled as DF/2021/289, has been deposited at the herbarium of the Medical Botany Chair of University of Salerno. Figure 2 reports a photo of the collected fresh purslane.

### 2.2. Purslane Samples Characterization

#### 2.2.1. Moisture Contents

Representative samples of purslane parts, both fresh and dried, underwent moisture content (*MC*) evaluations by using an IR Ohaus thermo-balance device (moisture analyzer mod MB45, Merck, Darmstadt, Germany). The *MC* is calculated, by incorporated software, as the water content on a wet basis (wb):

(1) $$MC,\%=\frac{wet\;material\;weight-dry\;material\;weight}{wet\;material\;weight}\cdot 100$$

The measurements were performed in triplicate; the results refer to the average values with the standard deviation SD.

In the following, for practical uses, the moisture content is also expressed as the moisture ratio (*MR*):

(2) $$MR=\frac{M-M_{e}}{M_{0}-M_{e}}$$

where M is the moisture content at any time, M_0_ is the initial moisture content, and M_e_ is the equilibrium moisture content; all are expressed as dried bases (db, water content on dry matter). The equilibrium moisture content of a material surrounded by air (as in a drying chamber) is the moisture content at which, without changes in conditions, the material is neither gaining nor losing moisture.

#### 2.2.2. Structural Macroscopic Observation, Pretratments, and Storage

Vegetal purslane structures are extremely heterogeneous (Figure 2), and this, from a technological point of view, constitutes a limit due to the different behavior of tiny parts (leaves, fruits, and seeds, with linear dimensions/thicknesses of a few millimeters), stems, twigs, and branches (length and thickness over centimeters) in conducting heat and mass transport during the drying process. In detail, purslane is glabrous, enlarged and fleshy, branched, smooth, hollow, and often reddish and it has prostrate-creeping stems of 10–40 cm. Its leaves, which are petiolate, are light green in color; they are also fleshy and glabrous like the stems, alternating with a spatulate form and truncated apex. In the axils of the leaves, solitary or in groups of 2–5, small and inconspicuous five-petal yellow flowers are present. These are open for a few hours in the morning (at sunny conditions). The fruits are compressed, fusiform, membranous capsules containing many seeds. These are tiny, black, glossy, oval-shaped seeds, about 1 mm in size, that can remain viable in the soil for 20 to 40 years. Seed germination occurs when the soil temperature exceeds 25 °C with an optimal temperature requirement of 30 °C [9].

In this study, spontaneous purslane was used. After its harvest, rough pretreatments were applied; these were in the form of inspections on extraneous matter and foreign matter as established by the European Spice Association—ESA—for herbs and spices [37]. Then, the collected plants, stored in shaded and airy conditions, were separated from the soil residues, and the thick branches were cut. These latter items are commonly waste products; in our study, these parts have been considered as possible sources of nutrients. Therefore, three groups of samples were prepared. The first one has included leaves and tiny twigs (samples MWD1-2-3); the second group includes the thick branches (samples MWD4t-5t-6t). In this way, more homogeneous structures have undergone drying protocols. Finally, the samples of whole purslane plant have been used in microwave-vacuum runs in a prototypal microwave apparatus (MWD7v). The sample codes and operative conditions are summarized in Table 1. It is important to note that this approach is closer to a possible industrial transposition of the drying treatments under investigation. Indeed, unlike several studies [12,38,39], purslane was not purchased at supermarkets where it can be already found cleaned and sorted into fine pieces, thus representing pre-treated and pre-selected products.

Treated samples were photographed to have a qualitative macroscopic comparison between fresh and dried products in terms of organoleptic properties (color changes/shrinkage).

### 2.3. Drying Treatments

Purslane samples were subjected to convective and microwave heating treatments. Table 1 summarizes the adopted drying protocols (sample codes and implemented operative conditions). Also, the fresh samples were included as controls.

#### 2.3.1. Convective Heat Supply

Convective drying at aerated shady conditions (CD1) was performed by positioning a layer of a few centimeters of purslane on a plane surface. Filter papers were used to cover the herb, which was left to dry at room temperature (25 ± 5 °C) for 5 days.

Hot-air convective drying (CD2 and CD3) was always carried out using a fresh material bed of a few centimeters placed on a suitable grid in a static, programmable electric oven (ISCO series 9000, Sabaudia-Latina, ProgiTec, Italy). Drying conditions applied: temperature 50 °C; time: 24 h (CD2) and 168 h (CD3). These conditions derive from the literature investigations [12,31].

#### 2.3.2. Microwave Heat Supply, Bench-Scale Investigations

Microwave-assisted drying was performed using a purslane bed on a grid sample holder that did not interact with the microwaves. The microwave apparatus for the bench-scale studies was a multimodal microwave cavity (2450 MHz—LBP 210/50 Microwave Oven 2300 W by InLand, USA) managed by a dedicated internal software (True-To-Power™ system to continuously vary the power supply). The apparatus was equipped in the basement with two modal stirrers to homogenize the electromagnetic field in the cavity. It is worth noting that the used multimode cavity allows us to modulate the power with a continued electromagnetic field emission, i.e., without pulsed modality, and to operate at power levels closer to industrial apparatuses compared with the commercial devices used in many literature reports.

During and/or at the end of the drying process, superficial temperature measurements of treated samples were performed by using an infrared thermometer (TASI TA601B, Entatial producer).

Several explorative radiating tests were carried out, changing the power level and time of exposure (process parameters). Through this preliminary work, the purslane–microwave interaction was estimated (thermal behavior under radiating operation) and thus the final (suitable) operative conditions were defined. In particular, as reported in Table 1, the selected power/time conditions were: 2300 W for 30 min (MWD1)—max power; 1150 W for 40 min (MWD2)—mild power; 460 W for 100 min—low power. Lots of 1 kg and 0.5 kg of fresh apical purslane and fresh purslane twigs were used, respectively.

#### 2.3.3. Microwave Heat Supply, Pre-Pilot-Scale Investigations

**Figure 3:**
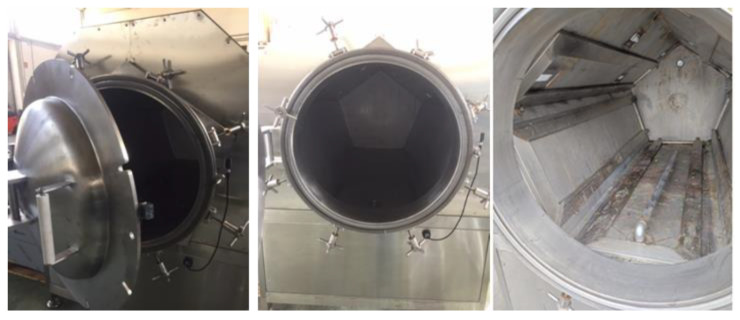
Photos of the microwave prototype.

With the aim of processing large amounts of purslane weed, the drying process was also conducted using a non-commercial pre-pilot microwave applicator. This pre-pilot apparatus was purposely designed and realized (EMitech Srl Company, Corato—Bari, Italy) to work at variable power levels and also in vacuum conditions due to the need to operate with herbs and spices, following the approaches of many literature studies [40,41]. In brief, the built-up pre-pilot apparatus presents a drying camera of 2 m^3^ built in stainless steel; it is equipped with 8 magnetrons at 1.5 kW (maximum power 12 kW) and a vacuum liquid ring pump (aspiration rate: 110 m^3^/h; vacuum guarantee: 940 mbar). Electromagnetic field homogenization is assured by the reflection phenomena promoted by the pentagonal section of the process camera. In Figure 3, several photos of the prototype are shown.

The prototype works as a batch system (herbs are manually loaded up grids in food-grade poly-propylene), and customized drying protocols can be implemented, varying several process parameters (managed via personal computer by a dedicated software) such as power, time, grade of vacuum, and load density (material amounts).

In this study, the preliminary results have been reported. They were achieved by using 5.5 kg of purslane as the batch load, and the operative conditions, selected after several preliminary investigations, were as follows: pressure—940 mbar; power level—7.5 kW for 10 min; 6 kW for successive 15 min; 4.5 kW for the final 20 min (code MWD7v Table 1). After the irradiation stages, before interrupting the vacuum, a pause of 5 min was conducted to promote a thermal levelling effect. Three tests were performed; the results, in terms of both qualitative and quantitative determinations, are shown as average evaluations/values.

#### 2.3.4. End Point of the Drying Activity

The end point of the drying activity was to obtain, for stabilizing purposes, a residual moisture content below 10% (wet bases, 0.11 on dried bases), a value that ensures the reduction of water activity (aw < 0.65) for a large variety of herbs and spices [37]. After drying, treated samples were photographed to obtain a qualitative macroscopic comparison between fresh and dried products in terms of color changes and shrinkage effects. Moreover, these observations, even if general and only qualitative, aimed to compare the achieved results in terms of the main organoleptic properties with those present in the literature.

### 2.4. Quality Profile of Dried Products

#### 2.4.1. Leaves Tissue Characterization

Drying processes can induce damage to the plant tissues through cracking and puffing effects. An indirect assessment of these effects can be performed by conductivity measurements due to increases in the rate of the release of ions (electrolytes) when certain quantities of plant structures are placed in a dissolution medium. In this study, electrolyte losses from purslane tissues to aqueous bulks were determined by the GLP31 conductometer (Crison, Spain). Small amounts of dried samples, about 0.30 g, were gently crushed by hand and placed in beakers containing water of known electrical conductivity (dispersion medium). After gentle stirring with a magnetic plate, the herb/water suspensions were subjected to electrical conductivity measurements at pre-established time intervals (measurements and sample storage at room temperature). The measurements were carried out after 1, 2, 3, 4 h; these times were chosen in order to observe detectable and significant increases in conductivity up to the final “saturation” values (24–78 h), for which the effects of cracking, puffing, and structural flaking, without distinction, contribute to electrolyte losses in the aqueous medium. The change in conductivity was reported as the difference between the conductivity measured at time *t* and the initial conductivity *t*_0_ (ΔC = C*t* − C*t*_0_). The results (in µS cm^−1^) are reported as the average values of at least three measurements with standard deviations SD. A one-way analysis of variance, ANOVA, was used to compare the results referring to the treated CD and MW samples (significantly different at *p*-value < 0.05; *p* > 0.05 similar values).

#### 2.4.2. Analytic Determinations

##### Extraction

The dried plant material was extracted in a Soxhlet apparatus for 10 days using petrol as solvent (40–65 C RPE—Carlo Erba Reagents—Cornaredo, Milan, Italy). The extracts were dried by using a Rotavapor^®^ instrument (BUCHI Italia S.r.l.—Cornaredo, Milan, Italy).

The yields (*w*/*w*, %) of the extracted compounds was calculated according to the following equation (dry mass basis was used in order to compare samples with different moisture content):

(3) $$Y\left(\%\right)=\frac{Weight\;Extract\;\left(g\right)}{Weight\;Dry\;Plant\;\left(g\right)}\cdot 100$$

##### Derivatization of Fatty Acids

Fatty acid methyl esters (FAMEs) were obtained by transmethylation following the method of El Riachy et al. 2019 [42].

##### GC/MS Analyses

Chromatographic separation was performed using an HP-5MS capillary column (30 mm, 0.25 mm, and 0.25 μm) and using helium as a carrier gas (1 mL/min). The injection (1 μL, 10% in *n*-hexane, *v*/*v* (Carlo Erba Reagents—Cornaredo, Milan, Italy) was carried out in split mode (50:1). The injector temperature was 250 °C, and the detector temperature was 180 °C. The ionization voltage, electron multiplier, and ion source temperature were set at 70 eV, 900 V, and 230 °C, respectively. The following elution program was used: 100 °C for 6 min, increased to 260 °C at 5 °C/min, and held at 260 °C for 30 min. The compounds were identified by calculating their Kovats retention index with respect to the reference standard available in our laboratories using the NIST 02 and Wiley 275 libraries [43].

##### Statistical Analysis

All tests were repeated three times. Data from each experiment are reported as the mean ± SD.

## 3. Results and Discussion

### 3.1. Dielectric Heating: Physical Issues

**Figure 4:**
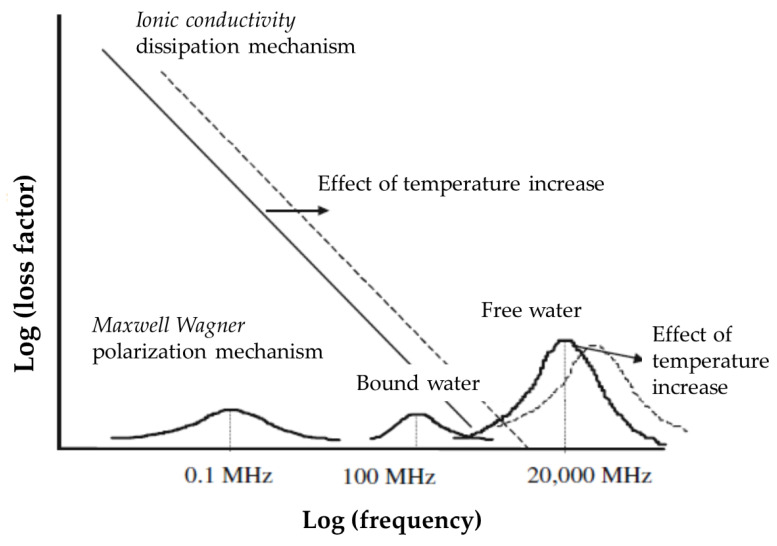
Qualitative representation of the dependence of the loss factor on the frequency and temperature variables (redrawn from [33]. The dashed lines emphasize the effect of temperature increase on the loss factor (dissipation capability).

As introduced, for agricultural purposes, microwave processing technologies have gained interest in postharvest operations for disinfestation, drying, and cooking treatments for use as proven, efficacious approaches to stabilize and prolong the shelf life of agro-foods (fruits, vegetables) that are susceptible to degradation due to the presence of natural infesting fauna [44,45] and enzyme activities. Moreover, the interest in this technology is promoted by the extensive research work on microwaving’s effects on food quality attributes in terms both of nutrients preservation and sensory features [32,40]. The success of the technology is due to the peculiar mechanism for energy transfer, which is particularly suitable for high-water content matrices such as agro-foods. In conventional heating processes, energy is transferred to a material by transport phenomena (convection, conduction, and radiation) that are driven by thermal gradients and through the external surface of treated materials. In contrast, microwave energy is directly delivered to materials through molecular interactions (named “loss mechanisms”; Figure 4) with an electromagnetic field via the conversion of electromagnetic energy into thermal energy (dielectric heating). When loss mechanisms take place, high rates of heating and efficiency of energy conversion are expected. These latter features represent the key point of microwave heating because the method makes it possible to accomplish in short times what would take a long time to accomplish with conventional heating (conductive and convective heat transfers). Indeed, this depends upon the slowness of the heat delivery rate from the material surface to the core as determined by the difference in temperature from a hot outside to a cool inside and by the thermal features of the materials (thermal conductivity and thermal diffusivity, which is in general very poor for the foods). In contrast, the use of microwave energy can allow, under some conditions (see penetration length), for bulk heating, with the electromagnetic field interacting with the material as a whole. The main results of this evidence are the possibility to reduce the heat degradation of materials and to reduce energy requests, making the thermal treatments performed by microwave heating intensified operations. It is important to highlight that process intensification (PI) is the modern industrial approach for developing equipment miniaturization and responsible–sustainable methods to enhance mass, heat, and momentum transfer rates to improve safety, scale-up procedures, and exploitation of resources, especially with respect to energy [46]. Electromagnetic interactions of energy–matter are due to the ability of the electric field to polarize elementary matter charges, and to the impossibility of the induced dipoles to follow the rapid changes of the oscillating electric field (thus, loss; or, more properly, dielectric dissipative mechanisms). Different kinds of polarization mechanisms are possible [33,35]; the dipoles rotation is the dominant polarization mechanism in irradiating materials rich in water (such as biological tissues, foods) in the microwave electromagnetic spectrum region (industrial high frequency heating 10^7^ < f[Hz] < 10^9^). Moreover, in the same frequency region, if ionic species are present in materials, further dissipative phenomena by ionic conduction (Joule’s loss effect) may occur.

The macroscopic matter features responsible for microwave heating are the dielectric properties (or permittivity), which express the energy coupling with the applied electromagnetic microwave field.

The main conclusions of the above-reported insights are that dielectric properties play a fundamental role in microwave heating. Vegetables/herbs and, more generally, plant matter/water mixtures are suitable materials for microwave heating technology due to their water contents and composition (free water, presence of ions with low polarity, non-polar long-chain hydrocarbons, large polar molecules [47,48]. In particular, dipolar polarization is the most significant loss mechanism; the conductivity mechanism improves the dissipation capacity (measurements (or estimation methods) of dielectric properties of plants and herbs can be found in [29,30,49,50]). The different effect of temperature on loss mechanisms guarantees a continuous efficient heating. Mass transfer rates are enhanced in turn due to the sudden increase in water diffusion through intracellular space (symplast) and extracellular (apoplast) vegetal compartments. The penetration depths of the plant matter/water mixtures can change considerably during dehydrations operations that, in turn, depend on the structure and dimension of the vegetal parts. All of this suggests that plants, or different parts of the same plants under microwave radiation, may exhibit different thermal behaviors, thus requiring investigations into suitable plane exposure conditions.

### 3.2. Insight Drying Treatments Results

#### 3.2.1. Dried Samples Observation

The performances of the applied drying protocols (codes and conditions are reported in Table 1) are summarized in Table 2. The drying rates were calculated considering the residual moisture content and the time required to achieve it.

#### 3.2.2. Apical Samples Drying

**Figure 5:**
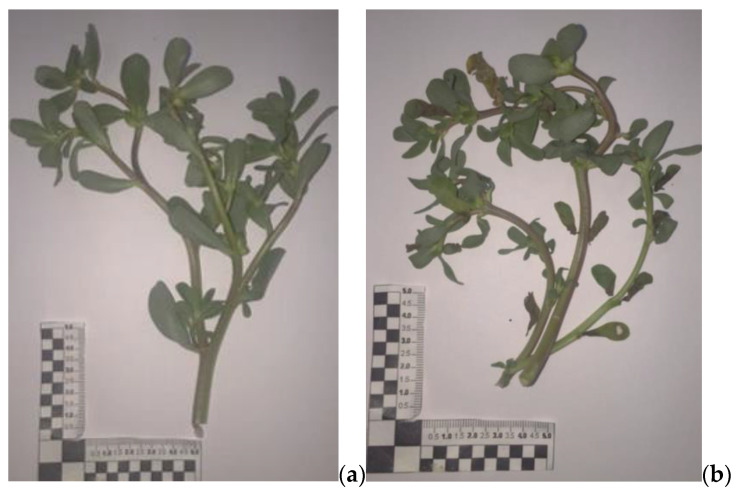

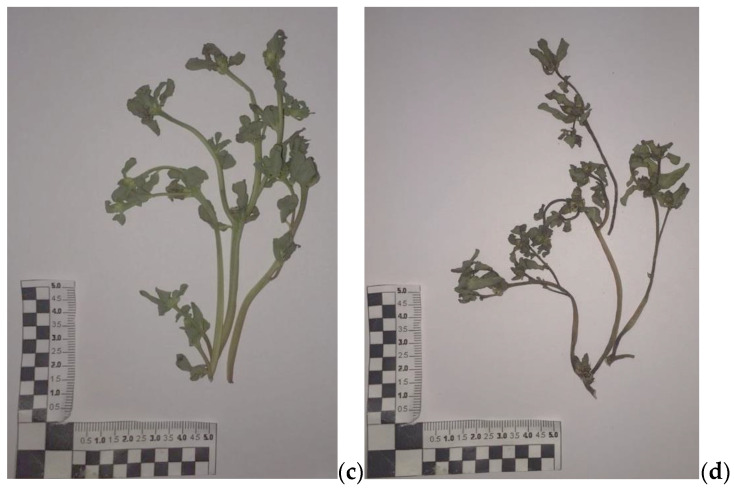
(**a**) Fresh purslane sample; (**b**) partially dried sample, CD1. Dried purslane samples at (**c**) 50 °C for 24 h, CD2, (**d**) and for 96 h.

**Figure 6:**
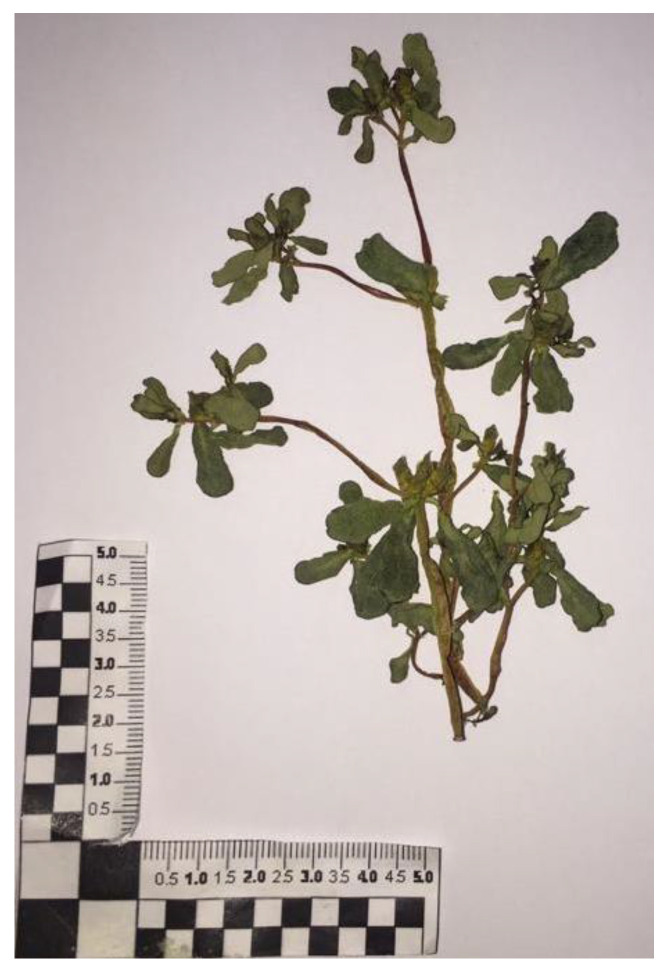
Microwave-dried purslane sample (MWD1).

Samples underwent convective treatments (CD1, shade drying and CD2, hot air drying) and have shown that, under the imposed conditions, the dehydration end points that are not suitable for a safe stability. In particular, purslane samples after several days at room condition manifested clear traces of degradation, including the development of infestants. When prolonged in a hot air (CD3) well, dried products were obtained. In Figure 5a–d and Figure 6, photos of fresh and dried purslane samples are presented to show, even if only as a qualitative manner, the shrinkage levels and color changes, confirming evidence in the literature for the hot-air drying of herbs [32].

In samples dried at ambient conditions (CD1), tissue shrinkage and color variation were very reduced due to the retention of most of the samples’ initial humidity after 5 days. Some small leaves of the apical parts began to darken (Figure 5b). Samples dried under hot air conditions (CD2 after 24 h—CD after prolonged time—96 h at 50 °C) visibly showed progressive and marked browning and tissue shrinkage effects (Figure 5c,d).

Samples dried via dielectric heating reached, as expected, a low moisture content in a shorter time (samples MWD1–MWD3). General and qualitative observations confirm literature evidence on better color retention for all vegetal parts [32,41]. This achieved result confirms experimental literature evidence for similar tests on purslane herbs [31,38]. Even if thermal stress affects compounds such as chlorophyll [48], the reduced times save this organoleptic feature. In our study, during microwave treatments, temperature peaks of 65.5 °C, 64.0 °C, and 57.3 °C were reached for a short time (time span in the order of a few minutes) when working at 2.3 kW, 1.15 kW, and 0.46 kW, respectively. Shrinkage effects were more extensive in samples dried at the maximum applied power (Figure 6). They are related to the drying rates that can encompass tissue puffing, cracking, and collapsing [28,30].

The impact of heating phenomena (convective vs. dielectric) on dried product features (structures and nutrients preservation) are reported in the following sections (*Tissue Integrity Evaluation* and *Chemical Investigations*).

**Figure 7:**
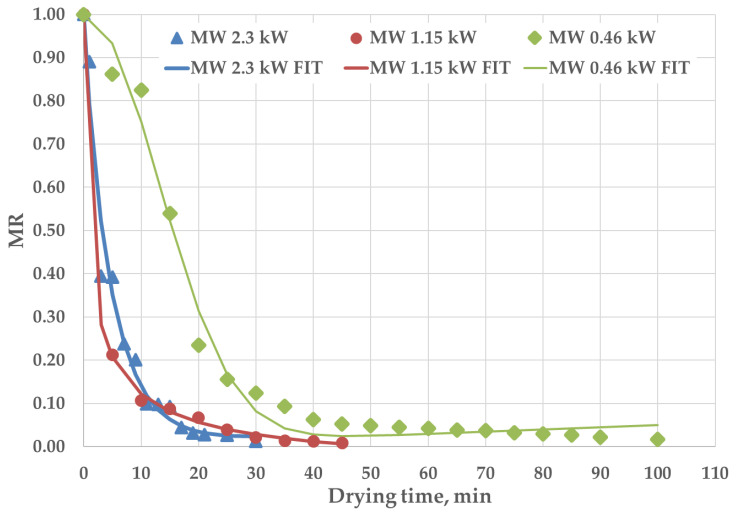
Drying curves at the explored microwave output powers. Solid lines represent best-fit lines using the Midilli–Kucuk model (each point is the mean of three replicates).

To study the effect of the microwave output powers on drying time, drying curve studies (moisture ratio MR vs. time) were performed. In Figure 7, the symbols represent experimental points, and the lines represent the Midilli–Kucuk model equation [51], the best fitting approach found in several literature works on drying kinetics studies of purslane leaves [38,52]. The moisture ratio was calculated using Equation (2), assuming the equilibrium moisture content to be zero (as in [52]); the modified Midilli–Kucuk model equation and associate parameters are summarized in Table 3. As expected, the higher the power level, the shorter the time. This behavior allows, in turn, for a better global retention of natural components of purslane, as described in the following.

The microwave output power had an important effect on the drying rate (velocity of water removal); the latter decreased continuously with the reduction of the applied power (Table 3). The moisture content of purslane is high during the initial drying process, which results in higher absorption of the microwave, increased product temperature and, consequently, a higher drying rate due to higher moisture diffusion (the drying rates change progressively with the power supplied, reducing up to 70% when proceeding from the maximum to the minimum power applied). In the achieved curves, no constant rate period was detected (the good performance of the Midilli–Kucuk model equation also demonstrates that one kind of law is enough to describe the moisture ratio vs. time trends). The results agree with those of [38,52] on the drying of purslane and various food products, respectively.

The drying rates of convective treatments (in oven at 50 °C) showed very low values (order of 10^−4^ kgH_2_O/min) due to the reduced mass transfer phenomena associated with the poor heat transport inside the vegetal tissues.

#### 3.2.3. Twigs Samples Drying

**Figure 8:**
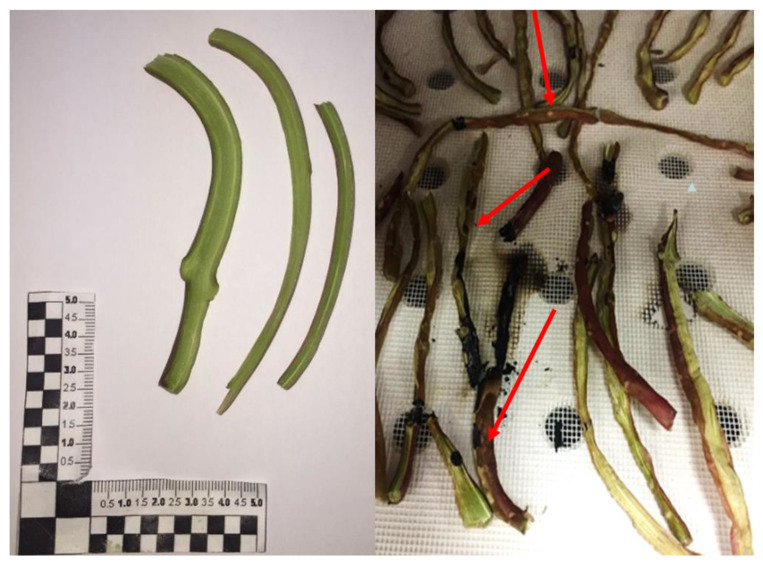
Purslane fresh-cut twigs (**on the left**); burned purslane twigs after preliminary drying test at 2.3 kW (**on the right**). The red arrows indicate the burn effects due to dissipation mechanisms.

As previously discussed, constituents and structures play a crucial role in microwave energy dissipation capability. In this work, we observed that the thickest purslane branches (coarse comminuted segments with a length of about 10–15 cm; Figure 8 on the left), which underwent microwave irradiation for several minutes at 2.3–0.46 kW, showed evident burns and sparks formations due to the presence of a hotspot on the surface of the samples (Figure 8 on the right).

**Figure 9:**
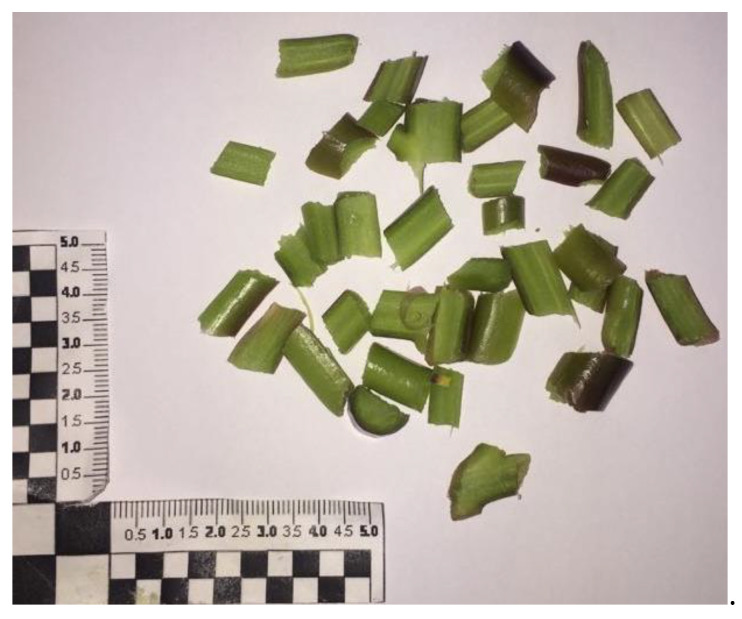
Purslane fresh fine-cut twigs.

This behavior did not surprise us; due to high humidity and mineral contents, the purslane branches have highly interacted with the electromagnetic field. Also, the effect of conductivity ions contributed to heat dissipation, and all this, along with the impossibility to quickly exchange the heat to the external environment, allowed for a sudden and localized increase in temperature and, in turn, burns. To promote faster external heat exchanges and thus avoid hotspot formation, purslane branches have been cut into small pieces (see Figure 9) to increase the samples’ superficial areas and thus heat and mass transport phenomena with their surroundings. In this way, the microwave treatments at different the three operative conditions have been successfully performed.

**Figure 10:**
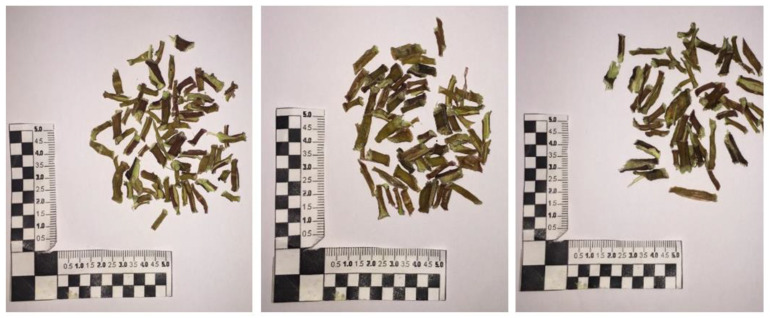
Purslane twigs after drying. From the left: at 2.3 kW—20 min; at 1.15 kW—30 min; at 0.46 kW—50 min.

It is worth noting that, generally, coarse twigs and branches are considered waste products and are not studied in the literature. From a technological point of view, these parts are difficult to process for edible purposes. Microwave drying allows us to obtain, after a minimal pretreatment, brittle structures that are easy to grind to produce purslane flours (Figure 10). As presented in the following, these parts of purslane are rich in fatty acids, sitosterols, and triterpenoids.

### 3.3. Tissue Integrity Evaluation

**Figure 11:**
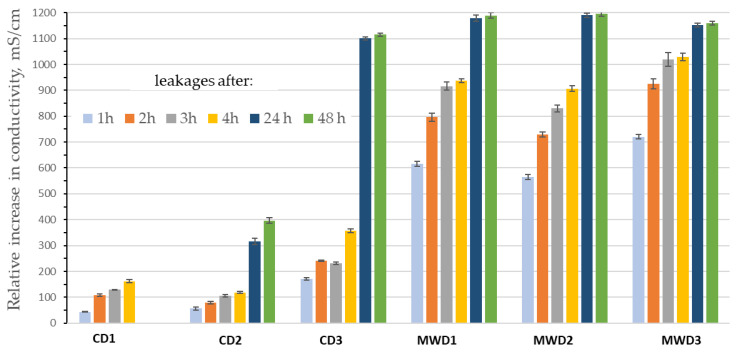
Relative increase in electrolyte losses from treated purslane samples (CD1—shade drying conditions; CD2—hot-air drying; MWD1—assisted microwave heating at 2.3 kW; MWD2—assisted microwave heating at 1.15 kW; MWD3—assisted microwave heating at 0.46 kW).

Cracking and puffing are the more diffuse effects encountered in vegetables, including in herbs dried by microwave-assisted treatments. Previous investigations [53,54,55] have demonstrated that these effects are attributable to the high rate of mass (liquid and vapor phases) transfer through the cell walls induced by the dielectric heating. A measure of structural damage after thermal stress can be evaluated through a conductometric assessment of the ionic conductivity. The efflux of substances from plants tissues submerged in liquid is widely used as a measure of vegetal membrane permeability [55,56]. Also, for the purslane dried samples, possible tissue alterations/ruptures have been investigated by an electrolyte loss determination method as previously performed for aromatic herbs [57,58,59]. In Figure 11, the electrical conductivities of the distilled water batch with purslane dried samples as a function of time are reported.

Drying at 50 °C for 24 h (CD2) involves the lower electrolytes leakages more so than the other drying protocols applied in this study (CD1–CD2, CD2–CD3, CD2–MWD1, CD2–MWD2, CD2–MWD3: *p* < 0.05) due to the relative short exposure time at 50 °C. In contrast, samples CD1 (shade drying) and CD3 (hot air for 7 days) show increasing losses values, even if for different reasons. The results for the CD1 sample can be reasonably interpreted in light of tissues with a still high moisture content that are present but are starting to release minerals due to the degenerative phenomena that have already begun in the structures due to exposure to room condition for 5 days (purslane is a very perishable herb). Instead, the electrolyte losses for the CD3 sample show the increase in damage due to prolonged exposure to hot air (50 °C for 7 days).

As expected, microwave treatments affect the tissue properties more strongly (MWD1–MWD2, MWD1–MWD3, MWD2–MWD3: *p* > 0.05). The increases in conductivity values are consistently different from those achieved for convective drying due to the action of dielectric heat dissipation and, consequently, the rate of mass transfer inside the tissues. Time of exposure and temperature peaks play a role on the leakages entity for the MWD1–3 samples. As highlighted for aromatic herbs [58], tissue desegregations are not necessarily drying drawbacks. In fact, they can facilitate or make selective other processes such as the extraction of nutrients/functional compounds [60], as found in the determination of the yields of the components extracted in this work (see next sections).

When prolonging all of the release tests until 24/48 h, it was observed that the conductivity of the aqueous bulks reached very similar plateau values (excepted for CD2; for CD1, data not available). These values can be defined from two effects. The first one is based on kinetics factors due to both the fast leakage from the apoplast compartment and the slow leakage from the symplast compartment. The second one is due to structural flaking.

### 3.4. Chemical Investigations

The chemical variability of the purslane samples has been recorded depending on both the plant part and treatment. In particular, all samples mainly consisted of fatty acids, albeit with different percentages, and in some cases, they also consisted of phytosterols. Furthermore, the extraction method can have significant effects on the fatty acid profile; thus, optimization and standardization of the extraction protocols are necessary to obtain comparable results [14].

The chemical composition of the aerial parts of purslane dried by convective and microwave methods is reported in Table 4. Chemical compositions of dried twigs and of whole dried purslane samples by the microwave under vacuum conditions are reported in Table 5 and Table 6, respectively. All of the reported percentage values refer to the dry mass of the samples.

**Figure 12:**
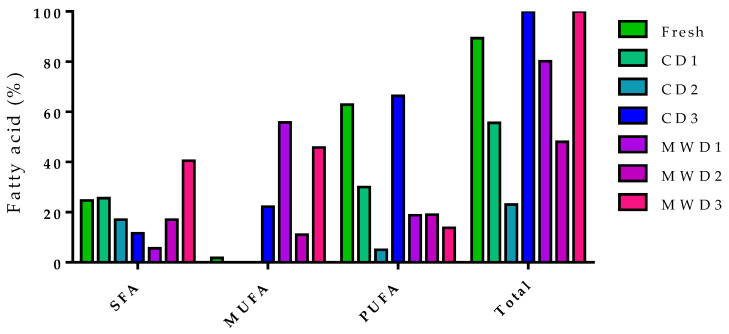
Fatty acids of fresh and dried purslane samples.

The drying process affected the chemical composition of the raw material (Table 4). The fresh sample was mainly characterized by fatty acids, which represented almost 90% of the extract (Figure 12). The major class of fatty acids were polyunsaturated fatty acids—PUFAs—(62.9%), followed by saturated fatty acids—SFAs—(24.7%), and finally monounsaturated fatty acids—MUFAs—(1.8%). The main component of the fresh plant extract was cervonic acid (58.9%). This compound was also reported by [61]. The second most abundant compound was palmitic acid, a saturated fatty acid, at 18.8%. In addition to fatty acids, phytosterols such as γ-sitosterol and β-sitosterol (2.4 and 0.7%, respectively) have also been identified.

The convective drying methods showed differences both between themselves and with respect to the fresh sample. In fact, in the case of CD3, the sample consisted exclusively of fatty acids, mainly PUFAs (66.4%) and MUFAs (22.2%). Linoleic acid was the most abundant component (48.3%), and this agrees with the available literature [7]. CD1 and CD2 samples were characterized by both fatty acids and phytosterols, whereas the MUFAs, which were found both in the fresh sample and in CD3, were absent. CD1 consisted predominantly of octacosanol (32.9%) and linoleic acid (20%), while CD2 consisted predominantly of ergost-5-en-β-ol, γ-sitosterol, and β-sitosterol (28.7, 26.3, and 21.4%, respectively).

Dielectric heating had a different impact on dried samples depending on the used power output, i.e., temperature–time conditions. In MWD1 and MWD3, fatty acids were the most abundant class of components, while in MWD2 fatty acids, they represented only 48% (Figure 12); the remainder was represented by sterols, in particular by γ-sitosterol (16.2%) and β-sitosterol (20.7%). These latter compounds were absent in MWD3 samples and were present in smaller quantities in MWD1. In fact, MWD1 was mainly characterized by oleic (54.12%) and linoleic acids (15%), while MWD3 was characterized by oleic (44.9%) and palmitic acids (17.6%). The reduced presence of α-linolenic acid compared with samples dried with convective methods agrees with the literature data [16,62]. MDW1 dried samples were subjected to the shortest treatment time at the highest temperature; the dried MDW3 samples were exposed to the lowest temperature range but for more prolonged times.

Consistent with the study of tissue integrity, the extraction yields were more abundant for the microwave-dried samples (both the apical and twigs parts; see Table 4 and Table 5, respectively). In particular, the highest applied power output (samples MWD1 and MWD4t) achieved the major percentage of valuable nutrients extracted. This result can be usefully exploited in the nutraceutical field, for example by promoting novel foods [63] based on the use of nanotechnologies (by polymeric and/or lipid-based platforms encapsulating the extracted nutrients/phytochemicals) [64,65]. Thus, microwave technology shows the best performance in terms of the recovery and preservation of the functional compounds of purslane.

**Figure 13:**
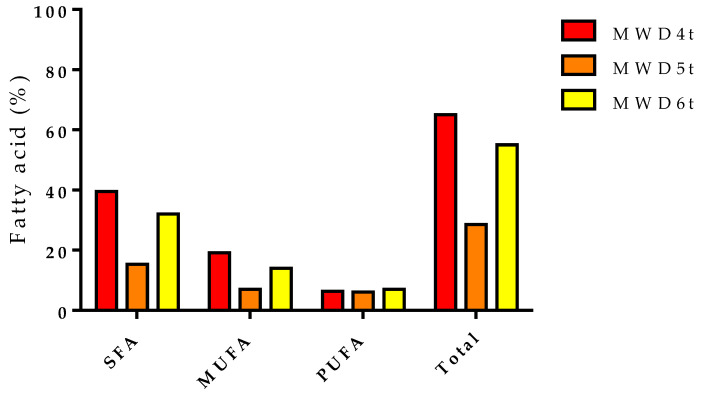
Fatty acids of dried purslane twigs.

Table 5 summarizes the characterizations of the twig samples dried by microwave protocols. The phytochemical analysis showed how, again, as for the apical purslane parts, the applied exposure conditions influenced the chemical composition. In MWD4t, fatty acids constituted 65%, where SFAs were the most abundant (39.5%), while MUFAs and PUFAs represented 19.1 and 6.4%, respectively (Figure 13). The saturated fatty acid that mainly characterized MWD4t was stearic acid (22.3%), while among the unsaturated fats, the most abundant was oleic acid (18.2%). In addition to fatty acids, phytosterols such as γ-sitosterol and β-sitosterol and triterpenes such as lupeol and botulin have been identified. In MWD5t, fatty acids represented only 28.5%, with stearic acid being the most abundant fatty acid (7.1%). In this case, the main components were octacosanol (14.8%) and β-sitosterol (28.9%); moreover, among triterpenes, α-amyrin was identified (5.7%). As far as MWD6t is concerned, however, among the various treatments, it is the one that has the greatest amounts of PUFAs (7.7%) compared with MWD5t (6.1%) and MWD4t (6.4%) due to the greater presence of α-linolenic acid (4.3%). Other fatty acids that characterized this sample were stearic (19.5%) and oleic acids (14.8%), while among the phytosterols, the main ones were γ-sitosterol and β-sitosterol (3.9 and 12.2%, respectively). Lupeol (9.1%) and α- (0.1%) and β-amyrin (2.3%) have been identified among triterpenes. No phytochemical literature data on the purslane twigs are available.

**Figure 14:**
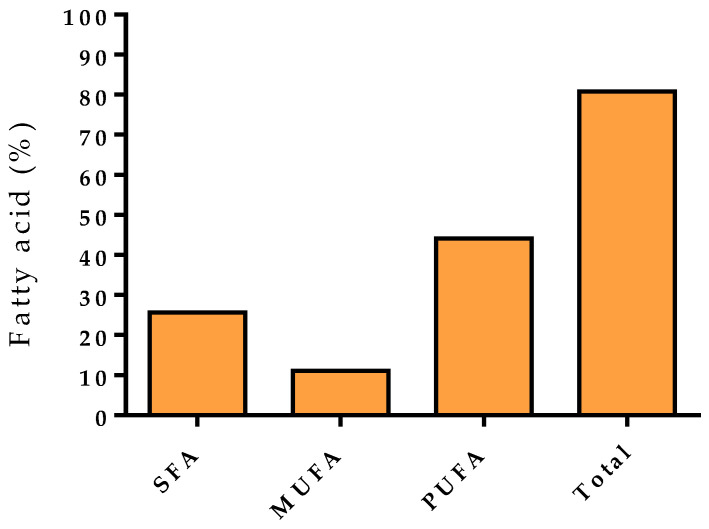
Fatty acids of microwave-vacuum dried purslane (MWD7v).

The use of a microwave-vacuum prototype confirms the interesting results already reported in the literature (color preservation, reduced shrinkage) [32]. Table 6 reports the chemical composition of the purslane extract dried using the prototype apparatus. Dried samples were mainly characterized by fatty acids (80.8%) (Figure 14), while phytosterols were present in low amounts. The main components were cervonic (27.8%—an omega-3) and palmitic acid (22.4%) which, on the other hand, is a saturated fatty acid. MUFAs were constituted exclusively by oleic acid (11.1%). These results emphasize the good potentiality of a microwave applicator for massive and easy to control productions, even if in batches. Currently, optimization studies are ongoing.

As summarized in the Introduction, purslane possesses a wide spectrum of pharmacological properties. Purslane’s high content in omega-3 fatty acids makes it a desirable component in nutritional protocol, preventing heart attacks and strengthening the immune system [66,67]. Indeed, due to its richness in ß-carotene and linolenic acid, purslane can be used as a health food for patients with cardiovascular disease [68] (α-linolenic cannot be synthesized in the human body, so it is important to take it through diet [15]). Also, the richness in phytosterols makes purslane a food worth valorizing. These compounds, which belong to the triterpene family, are similar to cholesterol in terms of function and structure. Phytosterols are naturally present in parts of all plants, and some researchers claim that they can promote human health when consumed regularly for a not too long period either in natural foods or in dietary supplements, lowering blood cholesterol levels [69].

## 4. Conclusions

In this study, *Portulaca oleracea* L. plants underwent drying treatments with the aim of developing a sustainable process to transform a widespread weed into a noble and health food source. For this purpose, spontaneous plants were collected from uncultivated land and used as a model matrix to investigate the impact and effectiveness of different drying treatments. Drying treatments for purslane, as for countless agro-food products, are required to prolong its shelf life and safety features during post-harvest storage.

The dielectric heating for drying purposes is characterized by a high matter–energy interaction; thus, short treatments times were requested due to the high rate of heat and mass transfer. The tissue structures of dried samples were compromised, and the extraction process was facilitated. Indeed, the extraction procedures present the highest yields of valuable compounds. Purslane microwave drying of apical parts of the weed allows us to obtain extracts rich in fatty acids —SFAs, MUFAs, and PUFAs— (more than 90% of components). High percentages of fatty acids were also achieved by the microwave-vacuum protocol in the entire purslane structure and in microwave-dried twigs, achieving percentages of 81% and roughly 65%, respectively. Phytosterols are less abundant; their highest preservation was found in microwave-dried twigs. The use of the microwave-vacuum prototype confirms interesting results, even if preliminary, especially for the treatable mass loads; however, to justify additional exercise costs for vacuum management, further studies are required.

The convective drying under the explored conditions (at room conditions; at 50 °C for short and prolonged times) guarantees structural integrity; however, the stabilization treatments appear scarcely convenient (or completely impracticable) from a technical point of view (prolonged time to reach a safety threshold of moisture content). Furthermore, the recovery of valuable components from dried samples appears to be globally less rich, in the form of the abundance and number of compounds both for fatty acids and phytosterols, than that obtained with microwave technology.

Undoubtedly, the dielectric heating, due to the peculiar interaction with vegetal constituents, can be a powerful tool for suitable massive post-harvest treatments of purslane. Its use can be seen as an advantageous, experienced methodology for achieving a fast-drying operation and better nutrient preservation.

## Figures and Tables

**Table 1:** Note on the applied drying protocols (samples code and selected operative conditions).

Protocol—Code	Drying Method/Operative Parameters
Fresh ^1^	Gentle manipulation after harvest; coarse screening to remove soil; storage at room conditions and in the shade
Convective Drying—CD1	Shade drying/Shady room conditions for 5 days
Convective Drying—CD2	Hot-air drying/Static oven at 50 °C for 24 h
Convective Drying—CD3	Hot-air drying/Static oven at 50 °C for 7 days
Microwave Drying—MWD1	Assisted microwave heating/2.30 kW for 30 min
Microwave Drying—MWD2	Assisted microwave heating/1.15 kW for 40 min
Microwave Drying—MWD3	Assisted microwave heating/0.46 kW for 100 min
Treated load:	≈1 kg
Microwave Drying—MWD4t ^2^	Assisted microwave heating/2.3 kW for 20 min
Microwave Drying—MWD5t ^2^	Assisted microwave heating/1.15 kW for 30 min
Microwave Drying—MWD6t ^2^	Assisted microwave heating/0.46 kW for 50 min
Treated load:	≈0.5 kg
Microwave Drying under Vacuum—MWD7v ^3^	Assisted microwave heating/7.5 kW for 10 min; 6 kW for further 15 min/4.5 kW for further 20 min
Treated load:	≈5.5 kg

^1^ Untreated product is indicated as fresh or raw material; ^2^ t refers to tick twigs, normally waste parts of purslane; ^3^ MWD7v refers to the entire purslane plant.

**Table 2:** Residual moisture content and drying rate measurements.

Sample Code	Final Moisture Content, % wb
Fresh apical parts	86.63 ± 2.51
Fresh twigs	91.69 ± 0.54
CD1	76.66 ± 2.17
CD2	71.93 ± 3.30
CD3	3.14 ± 0.68
MWD1	7.02 ± 2.40
MWD2	7.05 ± 0.91
MWD3	8.12 ± 2.47
MWD4t	8.43 ± 2.43
MWD5t	7.01 ± 0.53
MWD6t	8.79 ± 0.44
MWD7v	4.80 ± 2.00

**Table 3:** Midilli–Kucuk model equation, estimated coefficients, and statistical analysis; drying rate values.

Controlled Variable Parameters (Sample Code)	Drying Time, t, min	Final Moisture Content, M, db	Constants	R^2^	SSE	Drying Rate, kg_H2O_/min
2.30 kW (MWD4t)	30	0.076	k = 0.23396; *n* = 0.93517; b = 0.00067	0.9976	0.0303	0.2135
1.15 kW (MWD5t)	40	0.076	k = 0.80035; *n* = 0.41491; b= −0.00033	0.9994	0.0005	0.1601
0.46 kW (MWD6t)	100	0.108	k = 0.00277; *n* = 2.02366; b = 0.0005	0.9866	0.0265	0.0637

Modified Midilli–Kucuk eq.; MR = exp (−k t^n^) + b t [51]; in this study, the pre-exponential term was one.

**Table 4:** Chemical composition of fresh and dried purslane samples.

RT	Compound	Abundance%						
		Fresh	CD1	CD2	CD3	MWD1	MWD2	MWD3
22.587	Myristic acid	-	-	-	-	-	3.8 ± 0.4	9.2 ± 0.8
24.401	Palmitic acid	18.8 ± 0.8	-	-	10.5 ± 0.5	4.7 ± 0.3	7.7 ± 1.2	17.6 ± 1.9
24.794	Palmitoleic acid	-	-	-	-	-	-	0.9 ± 0.3
27.621	Stearic acid	-	-	-	-	-	1.5 ± 0.2	13.7 ± 0.7
27.754	Oleic Acid	1.8 ± 0.5	-	-	17.9 ± 1.6	54.2 ± 2.9	11.3 ± 0.5	44.9 ± 2.3
27.957	Linoleic acid	1.1 ± 0.6	20.0 ± 1.2	-	48.3 ± 2.6	15.0 ± 1.6	15.5 ± 0.9	8.9 ± 1.6
28.136	α-Linolenic acid	2.6 ± 0.8	-	-	6.7 ± 1.3	3.8 ± 0.5	1.3 ± 0.1	4.8 ± 0.8
31.575	γ-Linolenic acid	-	-	-	10.0 ± 1.6	-	-	-
32.028	Arachidic acid	1.9 ± 0.4	-	-	1.1 ± 0.3	0.9 ± 0.3	0.7 ± 0.2	-
34.236	Gadoleic acid	-	-	-	4.3 ± 0.6	1.6 ± 0.2	-	-
34.360	Behenic acid	4.0 ± 0.6	12.0 ± 0.9	7.3 ± 0.6	-	-	2.1 ± 0.2	-
37.220	Cervonic acid	58.9 ± 1.3	10.0 ± 0.8	5.7 ± 0.9	1.4 ± 0.2	-	2.6 ± 0.3	-
40.753	Lignoceric acid	2.4 ± 0.3	13.6 ± 1.1	10.6 ± 1.2	-	-	2.0 ± 0.8	-
45.769	Ergost-5-en-β-ol	4.1 ± 0.5	11.4 ± 0.9	28.7 ± 2.3	-	-	4.6 ± 0.6	-
46.120	Octacosanol	-	32.9 ± 1.5	-	-	5.0 ± 0.6	7.0 ± 0.8	-
46.501	Campesterol acetate	-	-	-	-	-	0.3 ± 0.1	-
54.590	γ-Sitosterol	2.4 ± 0.3	-	26.3 ± 2.1	-	-	16.2 ± 0.8	-
54.670	β-Sitosterol	0.7 ± 0.1	-	21.4 ± 1.9	-	14.7 ± 0.9	20.7 ± 0.9	-
	Yield (% *w*/*w*)	0.2	1.4	1.1	3.7	5.1	3.5	3.6

**Table 5:** Chemical composition of dried purslane twigs.

RT	Compound	Abundance %		
		MWD4t	MWD5t	MWD6t
24.401	Palmitic acid	12.8 ± 1.2	4.6 ± 0.5	10.1 ± 1.1
24.794	Palmitoleic acid	0.2 ± 0.1	-	-
27.621	Stearic acid	22.3 ± 1.8	7.1 ± 0.6	19.5 ± 1.8
27.754	Oleic Acid	18.2 ± 1.2	6.9 ± 0.6	14.8 ± 1.1
27.957	Linoleic acid	0.8 ± 0.2	3.0 ± 0.4	1.7 ± 0.3
28.136	α-Linolenic acid	3.2 ± 0.9	2.2 ± 0.6	4.3 ± 0.4
32.028	Arachidic acid	0.6 ± 0.2	0.1 ± 0.01	0.8 ± 0.2
34.236	Gadoleic acid	0.7 ± 0.2	0.1 ± 0.01	-
37.220	Cervonic acid	2.4 ± 0.6	0.9 ± 0.2	1.7 ± 0.5
40.753	Lignoceric acid	3.8 ± 0.8	3.6 ± 1.1	2.3 ± 0.9
45.769	Ergost-5-en-β-ol	1.6 ± 0.5	-	1.3 ± 0.3
45.804	β-Sitosterol acetate	-	2.8 ± 0.7	3.2 ± 0.9
46.120	Octacosanol	19.3 ± 1.8	14.8 ± 1.2	12.4 ± 1.6
46.501	Campesterol acetate	0.6 ± 0.2	-	-
50.458	Campesterol	-	5.6 ± 0.8	1.0 ± 0.3
50.773	Olean-13(18)-ene	-	1.3 ± 0.2	-
54.590	γ-Sitosterol	5.6 ± 0.8	4.3 ± 0.5	3.9 ± 0.6
54.670	β-Sitosterol	3.2 ± 0.6	28.9 ± 1.6	12.2 ± 0.9
55.397	α-Amyrin	-	5.7 ± 0.9	0.1 ± 0.01
55.635	β-Amyrin	-	-	2.3 ± 0.6
58.026	Lupeol	2.1 ± 0.9	7.9 ± 1.6	9.1 ± 1.3
58.560	Betulin	0.9 ± 0.5	-	-
	Yield (% *w*/*w*)	2.4	1.3	0.7

**Table 6:** Chemical composition of microwave-vacuum dried purslane samples.

RT	Compound	Abundance % MWD7v
24.401	Palmitic acid	22.4 ± 1.6
27.621	Stearic acid	2.3 ± 0.6
27.754	Oleic Acid	11.1 ± 0.7
27.957	Linoleic acid	11.8 ± 1.3
28.136	α-Linolenic acid	4.5 ± 0.9
32.028	Arachidic acid	0.9 ± 0.05
37.22	Cervonic acid	27.8 ± 0.6
54.59	γ-Sitosterol	9.3 ± 0.9
54.67	β-Sitosterol	6.0 ± 1.3
57.267	Propanoic acid. 2-(3-acetoxy-4.4.14-trimethylandrost-8-en-17-yl)	2.4 ± 0.9
	Yield (% *w*/*w*)	3.3

## Data Availability

The data used to support the findings of this study can be made available by the corresponding author upon request.
